# *Operando* photocatalytic cell for time-resolved XAS/GC analysis of gas phase CO_2_ photoreduction

**DOI:** 10.1107/S1600577525008768

**Published:** 2026-01-01

**Authors:** Sébastien Roth, Audrey Bonduelle-Skrzypczak, Christèle Legens, Julie Marin, Laurent Barthe, Anthony Beauvois, Valérie Briois, Pascal Raybaud

**Affiliations:** a IFP Energies Nouvelles Rond-Point de l’échangeur 69360Solaize France; b Synchrotron SOLEIL L’Orme des Merisiers 91190Saint-Aubin France; c Centre National de la Recherche Scientifique UR1 France; Advanced Photon Source, USA

**Keywords:** XAS *operando*, CO_2_ photoreduction, gas phase, Mo *K*-edge, XAS operando

## Abstract

This article reports an *operando* X-ray absorption spectroscopy (XAS) cell and its set-up for the photocatalytic reduction of CO_2_ in the gas phase. Details about the cell and set-up development and configuration are provided. The *operando* cell and set-up are validated from a photocatalytic point of view: the performances of two photocatalysts are successfully benchmarked with those obtained on an already existing gas phase photoreactor unit. The cell is also validated from an XAS point of view as measured Mo *K*-edge spectra of the photocatalyst loaded in the cell are similar to those measured for *ex situ* samples, prepared in a capillary. Finally, a detailed example of an *operando* photocatalytic experiment shows the potentiality of the developed cell to monitor photocatalysts under working conditions for gas phase CO_2_ photoreduction, making the link between a shift in the absorption edge due to Mo species evolution and the deactivation of the photocatalyst. Additionally, the developed *operando* cell, available at Synchrotron SOLEIL on the ROCK beamline, is versatile and may actually be used for any photocatalytic gas phase reaction.

## Abstract 2

This article reports a versatile *operando* X-ray absorption spectroscopy (XAS) cell and set-up for the photocatalytic reduction of CO_2_ in the gas phase, which is validated individually for both photocatalysis and XAS measurements. A detailed *operando* experiment reveals the link between Mo species evolution and deactivation of the photocatalyst.

## 1. Introduction

Reducing CO_2_ emissions to tackle global warming of the Earth is one of the major challenges of the century. Among the CO_2_ conversion methods: thermal, electrochemical, biochemical, *etc.* (Taheri Najafabadi, 2013 ▸; Kondratenko *et al.*, 2013 ▸), photocatalysis is appealing since it exploits energy from the sun, which is abundant [400 times higher than the annual energy demand (Lewis & Nocera, 2006 ▸)]. However, the photocatalytic conversion of CO_2_ is a complex process that still suffers from very low energy efficiency yield, well below the 10% targeted efficiency for at least 10 years (Lin *et al.*, 2022 ▸; Hisatomi *et al.*, 2015 ▸), hence it cannot be readily applied on an industrial scale (Roth *et al.*, 2025 ▸). The low efficiency may arise from many intricate physical and chemical limitations due to the materials, such as light penetration and absorption, charges recombination, CO_2_ stability, selectivity (competing hydrogen evolution reaction), nature of active sites, photocatalyst deactivation, *etc.* (Gong *et al.*, 2022 ▸). There is hence a difficulty in identifying the origins of these limitations, preventing an understanding and improvement of the photocatalytic materials. As a consequence, there is a clear need to develop new cutting-edge characterization tools to overcome these limitations.

In this sense, monitoring the photocatalyst under working conditions, including the evolution of the oxidation states of the metallic elements constituting the active phase, observation of reaction intermediates, identification of the active species and time-resolved quantification of products, could be very powerful to obtain a deeper comprehension of reaction mechanisms and structure–activity relationships of the involved species. *Operando* characterizations are expected to address these challenging questions in order to allow the design of more efficient photocatalysts. *Operando* Fourier transform infrared spectroscopy (FTIR), Raman, electron paramagnetic resonance (EPR) and X-ray absorption spectroscopy (XAS) have already been applied in the photocatalysis field (Issa Hamoud *et al.*, 2022 ▸). Regarding CO_2_ photoreduction, *operando* FTIR (Dankar, Pagis *et al.*, 2023 ▸) and *operando* diffuse reflectance infrared Fourier transform spectroscopy (DRIFT) (Wang *et al.*, 2021 ▸) have been previously reported. These IR characterizations provided relevant information on the intermediates formed during reaction, but it is more limited about the catalytic active phase evolution during photocatalysis. In this sense, considering its atomic selectivity, *operando* XAS is particularly interesting since it provides information on the local and electronic structures of the absorbing elements of working species (Bordiga *et al.*, 2013 ▸). *In situ* XAS has been reported for gas phase CO_2_ photocatalytic reduction (Liu *et al.*, 2017 ▸). *Operando* XAS has already been reported for liquid-phase photocatalytic CO_2_ reduction (Hu *et al.*, 2020 ▸) and water splitting (Tsyganok *et al.*, 2020 ▸), as well as gas phase photocatalytic reforming of methanol (Muñoz–Batista *et al.*, 2018 ▸) and ethanol dehydration (Piccolo *et al.*, 2020 ▸). However, to the best of our knowledge, *operando* XAS has never been reported for gas phase CO_2_ photocatalytic reduction applications. Therefore, the objective of the present work is to report on the developed *operando* cell and set-up, available at Synchrotron SOLEIL on the ROCK beamline. We assess the set-up in the case of TiO_2_ P25 supported molybdenum oxysulfide (MoO_*x*_S_*y*_), recently proposed as a potential photocatalyst (Roth *et al.*, 2025 ▸) allowing the oxidation state of Mo species to be analysed *in situ* in the course of CO_2_ photocatalytic reduction.

In Section 2, we present all technical information related to the photocatalytic systems, the cell design, the global set-up, photocatalytic conditions and the XAS/micro gas chromatography (µGC) data acquisition. The results section is divided into three different parts. In the first part, the cell performances as a photocatalytic reactor are validated. In the second one, the cell impact on the XAS measurements is evaluated. Finally, the third part details an example of an XAS *operando* photocatalytic experiment to show the potentiality of the developed cell to monitor photocatalysts under working conditions for gas phase CO_2_ photoreduction.

## 2. Experimental details

### 2.1. Materials

Mo oxysulfides nanostructures supported on TiO_2_ P25 (Sigma Aldrich) were synthesized by incipient wetness impregnation of an Mo alkoxide precursor on TiO_2_ P25 at 3%_wt_Mo. The subsequent sulfidation of the material was performed at 20°C or 350°C under an H_2_S/H_2_ gas mix (15%_mol_/85%_mol_) followed by a thermal post-treatment at 350°C under vacuum. The two different materials are denoted as 3%MoO_*x*_S_*y*_(20°C)/TiO_2_ and 3%MoO_*x*_S_*y*_(350°C)/TiO_2_.

### 2.2. Cell design and configuration

**Figure 1:**
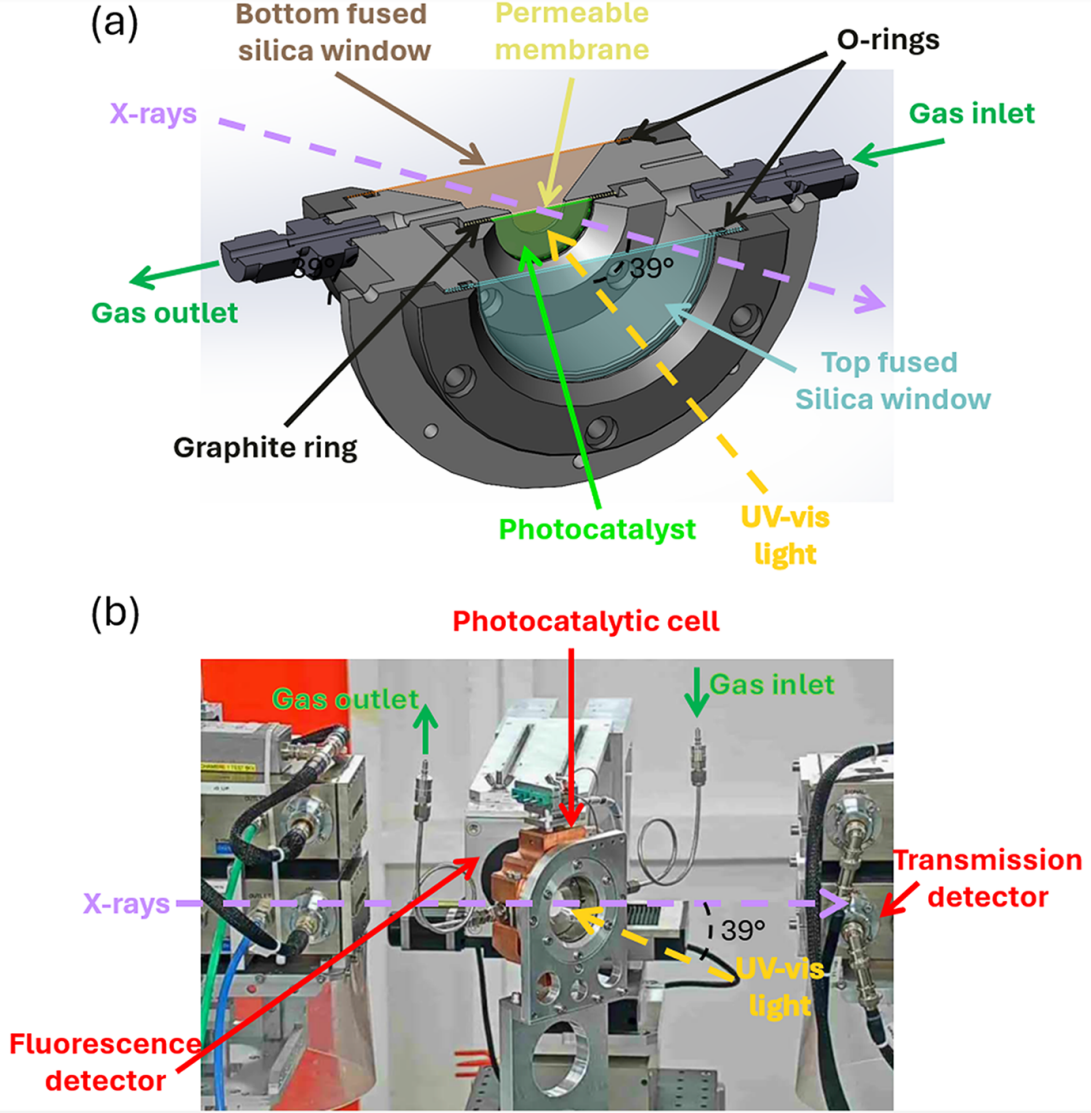
(*a*) Sectional schematic of the photocatalytic cell showing the different constituting pieces: fused silica windows, gas inlet and outlet, permeable membrane, graphite rings and O-rings. (*b*) Photograph of the *operando* XAS photocatalytic cell installed on the ROCK beamline.

The cell [Fig. 1 ▸(*a*)], allowing *operando* XAS to be performed on a working photocatalyst, is inspired by the reactor present at IFPEN (Dankar, Rouchon *et al.*, 2023 ▸) and consists of a stainless-steel cylindrical piece, whose total volume is 56 mL, embedded in a copper heated element equipped with two thermocouples. One is within the copper heating collar, close to the heating element, used to manage the temperature control. A second one lies within the cell body, very close to the inner volume wall, at the sample height, to measure the closest temperature to the inner chamber one. While the heating design allows to go higher, the cell is usable up to 200°C with respect to the Viton O-rings’ temperature limitation. A gas inlet on the top part of the cell and a gas outlet in the opposite bottom part allow gases to be introduced in passing-through mode (*i.e.* forcing the gas to cross the photocatalytic bed). Photocatalyst powder is gently pressed in the centre of the cell (surface of 3.8 cm^2^) on an inert binder-free glass microfiber membrane permeable to gases and transparent to X-rays (32 mm, Avantor, VWR Distribution). This membrane is held by two graphite rings (0.5 mm thickness and 2.2 cm internal diameter) and a metallic flange holding all together. The cell is hermetically closed by greased Viton O-rings and fused silica optical windows on both sides, which are transparent to UV–visible light and whose thickness can be adapted depending on the photon energy in order to keep the absorption of X-rays by the windows to a reasonable level with a minimum thickness of 170 µm for operating reasons (see Fig. S1 of the supporting information). Note that a Kapton back-window could possibly be used (minimum thickness of 25 µm) to further improve transmission. An optical fibre holder is installed on the top of the cell and allows the light source to be positioned perpendicular to the photocatalyst at a chosen distance.

The cell (total angular aperture of 90°) is mounted vertically in the set-up and tilted by 39° compared with the source of X-ray radiation, thus allowing the light source to be positioned out of the X-ray beam path, while being still mechanically perpendicular to the surface of the photocatalyst [Fig. 1 ▸(*b*)]. The back of the cell is placed in front of the X-ray source and the transmission detector is aligned with the source in the front part of the cell. A fluorescence detector is also placed in the environment of the cell to detect the fluorescence signal from the back of the cell. All the tilting angles of the cell can be adjusted. The optimized configuration is presented in Fig. 1 ▸(*b*).

Additional schematics of the *operando* cell are present in Section S2 of the supporting information.

### 2.3. Global set-up

**Figure 2:**
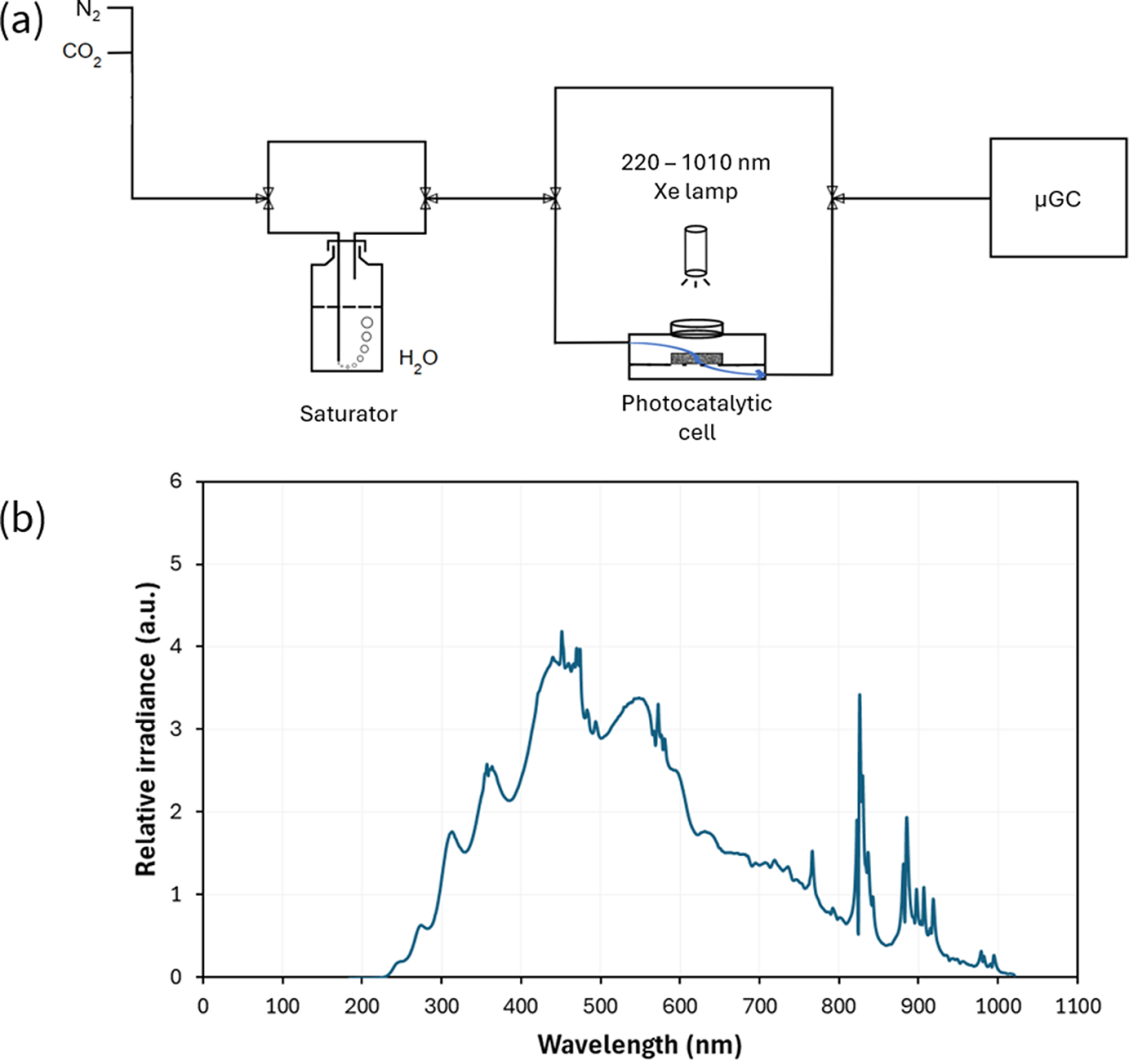
(*a*) Simplified scheme of the photocatalytic part of the set-up. (*b*) Irradiance spectrum of the LC8 Hamamatsu xenon lamp, measured with a SPECORD S 600 spectrometer from Analytik Jena.

A schematic illustration of the photocatalytic part of the set-up is represented in Fig. 2 ▸(*a*). Gas cylinders of 99.998% CO_2_ and 99.9999% N_2_ (supplied by Air Liquide) are further purified by O_2_ filters (Agilent) and used as feeding gases in the unit. Gases can be humidified by passing through a saturator (stainless-steel recipient of 270 ml containing liquid water). The photocatalyst is placed in the *operando* photocatalytic cell (previously presented in detail) and illuminated by a Hamamatsu LC8 Xenon lamp, reproducing the Sun irradiance spectrum from 220 to 1010 nm [Fig. 2 ▸(*b*)]. The emitted light is driven to the cell with an optical fibre. Finally, photoproduced gases are entailed by the original flux to a Fusion Inficon µGC for analysis.

### 2.4. *Operando* photocatalytic conditions

*Operando* XAS photocatalytic tests were performed at Synchrotron SOLEIL on the ROCK beamline, in the cell previously described, equipped with 200 µm- and 500 µm-thick fused silica windows at the bottom and at the top, respectively. In a typical test, 60 mg of photocatalyst in powder form is homogeneously packed in the cell, inside of a glovebox (due to the air sensitivity of the photocatalysts), to have a smooth and regular surface (3.8 cm^2^). Once the cell is connected to the set-up, the testing procedure is divided into two steps. The first step, the purge, consists of flushing the cell for 2 h with 8 ml min^−1^ of wet CO_2_ at *T*_saturator_ = 20°C (H_2_O ≃ 2.4%_vol_, *i.e.* CO_2_/H_2_O ≃ 40 mol mol^−1^), *T*_unit_ = 30°C and *P*_unit_ = 1.0 bar (working conditions parameters). The second step, the photocatalytic test, consists of passing 0.3 ml min^−1^ (3.1 h residence time in the cell) of wet CO_2_ with *T*_saturator_ = 20°C, *T*_unit_ = 30°C and *P*_unit_ = 1.0 bar. The photocatalytic bed is illuminated by the Xenon lamp with continuous or alternating (180 s ON and 180 s OFF cycle) UV–visible irradiation with a power of 80 W m^−2^ {in the 315–400 nm measuring range of the luxmeter [radiometer equipped with a CCD captor (Delta Ohm)]}. Gases analyses were carried out using a micro gas chromatograph equipped with a thermal conductivity detector (µGC-TCD) every 10 min and X-ray absorption spectra were recorded during the whole process, with one spectrum every 250 ms.

### 2.5. XAS and µGC data acquisition

Quick-XAS was performed on the ROCK beamline at Synchrotron SOLEIL (Briois *et al.*, 2016 ▸). X-ray absorption spectra at the Mo *K*-edge (∼20 keV) were recorded at a current of 450 mA in the storage ring, using an Si(111) channel-cut monochromator with a Bragg angle set at 5.5° and an oscillation amplitude of 0.5° (19800–21200 eV). The oscillation frequency was fixed at 2 Hz leading to a spectrum acquisition with increasing Bragg angles every 250 ms. Harmonic rejection was ensured using mirrors with Pd stripes aligned at 2.8 mrad with respect to the pink and monochromatic beam. Measurements at the Mo *K*-edge were performed in transmission mode using ionization chambers (Oken) filled with a mixture of 50% nitro­gen and 50% argon and in fluorescence mode using a passivated implanted planar silicon detector (Mirion technologies). A molybdenum foil was placed between the second and the third ionization chambers to serve as a reference for further energy calibration [calibrated at 20003.9 eV (Reschke *et al.*, 2013 ▸)]. Precautions to avoid any evolution of the Mo species induced by X-ray radiation were taken: the beam was defocused (size of 1.3 mm × 0.66 mm) and attenuated by a 2 mm-thick glass at the entrance of the first ionization chamber measuring *I*_0_, and the analysed area of the sample was changed every 30 min (with a remotely controlled motor moving the cell) throughout the test. An example of Mo spectra evolution when such precautions are not applied is provided in Section S3 of the supporting information.

The collected spectra were processed through a graphical user interface developed at Synchrotron SOLEIL and dedicated to the data obtained at the ROCK beamline. By using the ‘extract_gui’ module (Lesage *et al.*, 2019 ▸), the data were extracted on a common energy grid before being merged to improve the signal-to-noise ratio by averaging 30 consecutive spectra corresponding to 15 s of acquisition. Then, the ‘normal_gui’ module was used (1) to calibrate the average spectra on absolute energy scale relative to the first derivative of the Mo foil, recorded simultaneously, to the literature value at 20003.9 eV for Mo (Reschke *et al.*, 2013 ▸), and (2) to normalize the data on the atomic absorption using the same parameters for pre-edge polynomial background extraction (−200 to −60 eV, degree 1) and post-edge polynomial fitting (+60 to +1200 eV, degree 2).

Gases were analysed on-line by a Fusion Inficon µGC-TCD every 10 min during the photocatalytic test. The µGC-TCD is composed of two different columns, A and B, that are used to detect different compounds. Column A (molecular sieve, Ar carrier gas) was used to detect (with retention times in parentheses): H_2_ (81 s), O_2_ (98 s), N_2_ (112 s), CH_4_ (141 s) and CO (159 s). Column B (capillary, Ar carrier gas) was used to detect (with retention times in parentheses): CO_2_ (58 s), H_2_O (100 s). The µGC-TCD was calibrated with a gas cylinder containing 99.44%_mol_ CO_2_, 100 ppm_mol_ H_2_, 100 ppm_mol_ CH_4_ and 200 ppm_mol_ CO.

## 3. Results and discussion

### 3.1. Validation of the developed cell from a photocatalytic point of view

The photocatalytic performances of the cell and associated set-up developed on the ROCK beamline are first evaluated under continuous UV–visible irradiation (procedure detailed in Section 2.4) to be compared with the photocatalytic reactor and set-up commonly used in the laboratory of IFP Energies Nouvelles (IFPEN) (Dankar, Rouchon *et al.*, 2023 ▸) for two photocatalysts: 3%MoO_*x*_S_*y*_(20°C)/TiO_2_ and 3%MoO_*x*_S_*y*_(350°C)/TiO_2_. Since the illuminated surface of the photocatalyst on the ROCK and IFPEN set-ups are 3.8 cm^2^ and 7.1 cm^2^, respectively, the evolutions of the formation rates are reported in µmol h^−1^ m^−2^ so that they can be directly compared.

**Figure 3:**
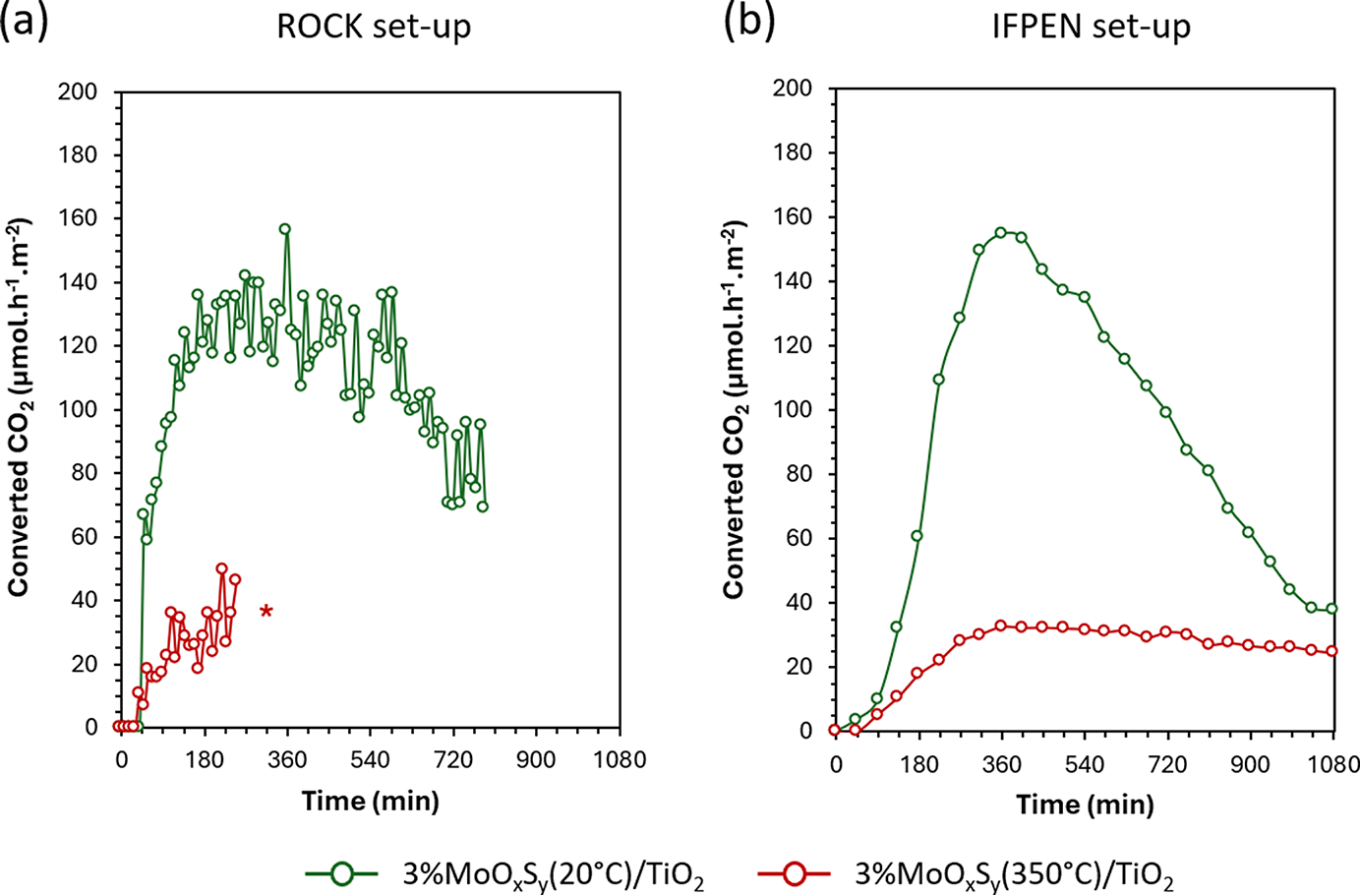
Converted CO_2_ (in µmol h^−1^ m^−2^) evolution in the (*a*) ROCK set-up and (*b*) IFPEN set-up for 3%MoO_*x*_S_*y*_(20°C)/TiO_2_ (green) and 3%MoO_*x*_S_*y*_(350°C)/TiO_2_ (red) under continuous UV–visible light irradiation. The red star means that the test was conducted with alternating UV–visible irradiation (as described in Section 2.4).

On both set-ups, the electronic selectivity towards carbonaceous products is higher than 80%, *i.e.* the competing hydrogen evolution reaction (producing H_2_) proceeds with an electronic selectivity below 20% (Section S4 of the supporting information). Among carbonaceous products, CH_4_ is the major electronic product, *i.e.* the product that consumed the most electrons to be formed (Section S4 of the supporting information). The evolution of converted CO_2_ (CO + CH_4_ formation rate) is similar between the two set-ups for a given photocatalyst (Fig. 3 ▸) and follows a bell shape curve: (1) increase of the formation rate up to a maximum in the first hours (experimental artefact due to the dead volumes as detailed in Section S5 of the supporting information), and (2) decrease of the formation rate due to the photocatalyst’s deactivation. Moreover, materials show photoreduction activities of similar order of magnitude, respecting their relative activity ranking.

Note that on the ROCK set-up, 3%MoO_*x*_S_*y*_(350°C)/TiO_2_ was not evaluated under continuous light irradiation but only under alternating light irradiation, which should not impact drastically on its performances. At maximum a factor of two on the activity should be observed between the two modes (Section S6 of the supporting information). Also, noisier data for the ROCK set-up may come from the smaller irradiated surface (3.8 cm^2^ < 7.1 cm^2^) and/or the sensitivity of the µGC-TCD (different in the two set-ups).

Overall, these results validate the cell’s reliability from a photocatalytic point of view since the performances of two different materials are consistent with the performances reported on a similar set-up at IFPEN.

### 3.2. Evaluation of the developed cell impact on XAS measurement

**Figure 4:**
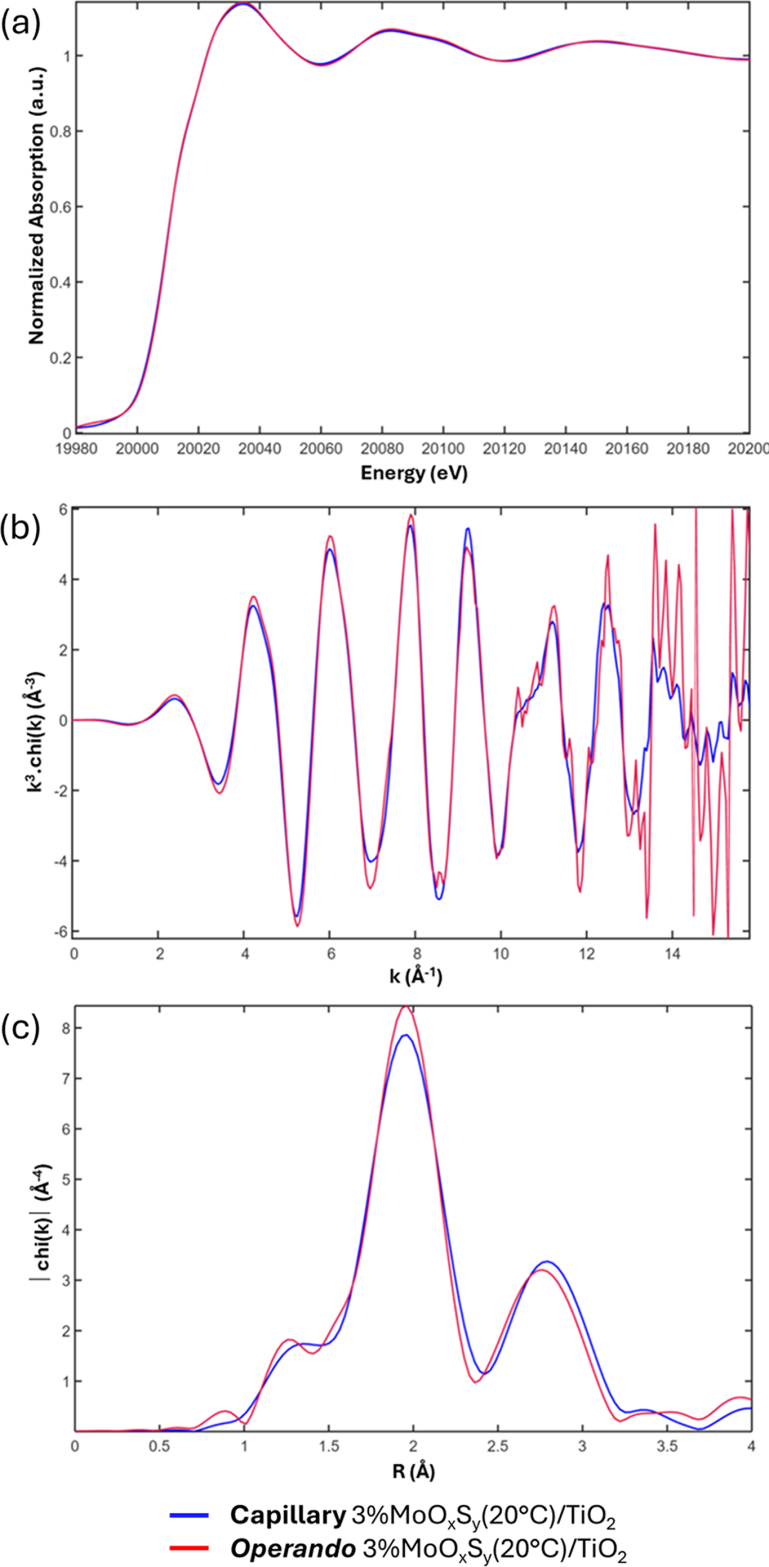
(*a*) Normalized transmission Mo *K*-edge XANES, (*b*) EXAFS [*k*^3^-weighted *χ*(*k*)] and (*c*) Fourier transform [*k*^3^-weighted χ|(*k*)| with *k* = 3.6–14.5 Å^−1^, d*k* = 2, Kaiser window] with *R* the non-phase-shift corrected radial distance for 3%MoO_*x*_S_*y*_(20°C)/TiO_2_ measured *ex situ* in a capillary (blue) and inside the XAS *operando* photocatalytic cell under N_2_ (red).

It is important to know whether the *operando* cell does not cause too much disturbance for the XAS analysis. To check this, we have chosen to compare the signal of 3%MoO_*x*_S_*y*_(20°C)/TiO_2_ (before test) powder inside a closed capillary and the one of the same powder inside the XAS *operando* photocatalytic cell under N_2_ flow. Fig. 4 ▸ reveals that the spectra are quasi-superimposable. However, the signal recorded inside the *operando* XAS photocatalytic cell appears noisier even though approximately the same quantity of scans is accumulated (∼3000 scans), certainly because, in this case, X-rays have to cross both a more important total thickness of glass to reach the transmission detector [∼700 µm (in *operando* cell) > ∼200 µm (capillary)] and a smaller thickness of the material which reduces the signal-to-noise ratio [∼1 mm (in *operando* cell) < ∼3 mm (capillary)]. Indeed, the recorded edge jump in the *operando* cell was about 0.03, compared with 0.4 in the capillary. This difference does not match the difference in thickness of material crossed by X-rays, but it may arise due to several factors: different density in the capillary compared with the cell, imprecise thickness evaluation in the cell, *etc.* Overall, the developed *operando* cell appears suitable for performing XAS measurements on photocatalysts.

### 3.3. Detailed *operando* XAS experiment for 3%MoO_*x*_S_*y*_(20°C)/TiO_2_

In this section, we will focus on the 3%MoO_*x*_S_*y*_(20°C)/TiO_2_ photocatalyst to illustrate the full *operando* XAS capabilities of the cell. This material was chosen as it is the most active among the two presented in this article. Only CH_4_ production will be considered in this section as no link could be drawn between CO production and XAS spectra evolution (Section S7 of the supporting information). Note also that *operando* experiments are carried out with alternating light irradiation (and not continuous) with the purpose that XAS data could be further processed with phase-sensitive detection (PSD) mathematical treatment to extract the contribution of only active Mo species (Urakawa *et al.*, 2023 ▸). This PSD analysis, beyond the scope of the present article, will be presented in a forthcoming paper.

**Figure 5:**
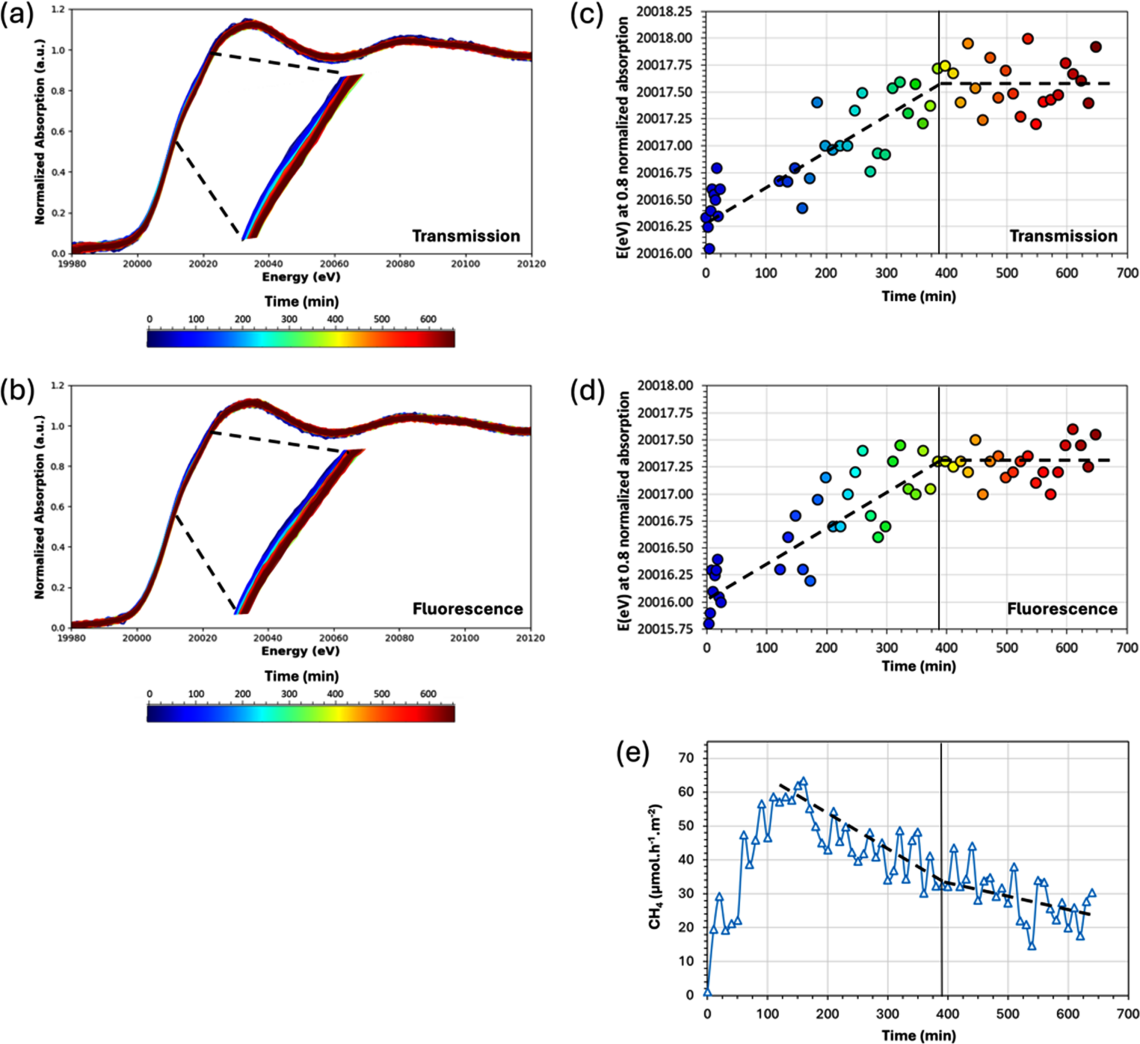
3%MoO_*x*_S_*y*_(20°C)/TiO_2_ normalized Mo *K*-edge XANES accumulated in (*a*) transmission and (*b*) fluorescence throughout the whole *operando* photocatalytic test. Energy (in eV) of (*c*) transmission and (*d*) fluorescence spectra at 0.8 of normalized absorption evolution in time, which is the region of the spectra which appears to evolve the most. (*e*) CH_4_ formation rate evolution (in µmol h^−1^ m^−2^). Dashed lines are a guide to the eyes to observe trends (not based on mathematical fitting).

The plot of the X-ray absorption spectra as a function of time (Fig. 5 ▸) reveals a slight evolution of the Mo species at the Mo *K*-edge throughout the photocatalytic test. Both transmission [Fig. 5 ▸(*a*) and Section S8 of the supporting information] and fluorescence [Fig. 5 ▸(*b*) and Section S8 of the supporting information] XANES spectra show small differences. This comparison shows that, thanks to the small thickness of the photocatalytic bed (∼1 mm), it is possible to probe surface phenomena occurring during photocatalysis, even with a rather bulk-sensitive technique like XAS. Indeed, fluorescence spectra, registered from the back of the cell, and transmission spectra show the same evolution during the photocatalytic test (Fig. 5 ▸), further emphasizing that the working part of the photocatalytic bed (the volume exposed to light) is not negligible.

Transmission and fluorescence spectra show that the absorption edge, around a normalized absorption of 0.8, reveals a shift toward higher energies (no data available between 30 and 120 min due to a beam dump). The observed shift in energy is +1.2 eV between the beginning and the end of the analyses [Figs. 5 ▸(*c*) and 5 ▸(*d*)]. Note that at half normalized absorption the energy increases as well from 20009.5 eV to 20010.3 eV during the *operando* XAS photocatalytic test. This shift reflects a change of the electronic structure of Mo with empty antibonding *p* Mo orbitals where the 1*s* electron is ejected to higher energies at the end of the photocatalytic test compared with the pristine materials. This shift can hence be linked either to a slight oxidation of the Mo species or to a structural change in the first coordination shell of the Mo such as a slight decrease of the metal–ligand distance [due to a change of the ligand nature (S to O) or to a reorganization (di­sulfido to sulfido)] (Schrapers *et al.*, 2015 ▸; Plais, 2017 ▸). The changes observed on the XANES spectra are also slightly reflected on the EXAFS spectra (Section S8 of the supporting information), which may indicate that the local geometry around the Mo atoms is affected during the photocatalytic test as well.

Note that the analysed position in XAS is changed every 30 min [and X-ray intensity is attenuated (Section 2.5)], therefore the mentioned changes are not due to beam damage, otherwise a shift toward lower energies at 0.8 normalized absorption should be observed when changing to a new position that has never received X-rays before, which is not the case (Section S3 of the supporting information).

In this experiment, the shift in energy is mainly occurring in the time interval between 0 and 385 min. This time interval, which corresponds to gases detection on the µGC-TCD at approximately the same time [almost simultaneous detection (Section S5 of the supporting information)], relates to the CH_4_ rate of formation curve when the deactivation is the steepest [Fig. 5 ▸(*e*)], not considering the evolution before reaching the maximum rate of formation which is only an artefact due to the process (Section S5). Oppositely, the deactivation is slower at times longer than 385 min and the energy at 0.8 normalized absorption is approximately constant.

From these observations, it can be hypothesized that the evolution of the Mo species is responsible for the first (and main) deactivation of the photocatalyst (time < 385 min), while the second one (slower) would mainly or even only be due to TiO_2_, since Mo species no longer evolve (time > 385 min).

## 4. Conclusion and perspectives

This article has presented the development of an *operando* XAS photocatalytic cell for reactions in the gas phase that was first validated from a photocatalytic point of view comparing the activity of Mo/TiO_2_ based photocatalyst used for CO_2_ photoreduction with results obtained in a cell that has already been tried and tested at IFPEN laboratory. Additionally, Mo *K*-edge XAS data recorded inside the cell and outside the cell (*ex situ* data) coincide for the same photocatalyst, which confirmed that the cell does not disturb the spectra.

A detailed example of an *operando* experiment highlighted the scope of data that can be obtained with the developed set-up and the relevance of XAS to probe surface phenomena happening during photocatalysis. The studied photocatalyst exhibited an Mo *K*-edge absorption edge shift by +1.2 eV during the photocatalytic test which correlates with the first deactivation stage of the photocatalyst. Therefore, it could be hypothesized that the evolution of the Mo species (oxidation state or structural change) is responsible for the first (and main) deactivation stage of the photocatalyst, while the second one would only be related to TiO_2_. This first level of data treatment will be further exploited with the PSD methodology to provide a deeper understanding of the working behaviour of the photocatalyst in a forthcoming paper.

Moreover, it is worth noting that the developed cell is versatile and could be applied to other gas phase photocatalytic reactions and be coupled to other product characterization techniques like mass spectrometry or Raman spectroscopy. Also, thanks to the adaptative type (SiO_2_ or Kapton) and thickness of the cell windows, elements other than Mo could be probed, even at a lower energy edge.

## Supplementary Material

Supporting Table S1 and Figures S1 to S9. DOI: 10.1107/S1600577525008768/vy5043sup1.pdf

## Data Availability

Within the article and as published supporting materials.
