# Supplementary material for: Operando photocatalytic cell for time-resolved XAS/GC analysis of gas phase CO2 photoreduction
Source: J Synchrotron Radiat. 2026 Jan 1;33(Pt 1):123–9. doi: 10.1107/S1600577525008768 (PMC12809429; doi:10.1107/S1600577525008768)
Supplement: Supplementary file 1 [file s-33-00123-sup1.pdf]

JOURNAL OF SYNCHROTRON RADIATION

Volume 33 (2026)

Supporting information for article:

Operando photocatalytic cell for time-resolved XAS/GC analysis of gas-phase CO2 photoreduction

Sébastien Roth, Audrey Bonduelle-Skrzypczak, Christèle Legens, Julie Marin, Laurent Barthe, Anthony Beauvois, Valérie Briois and Pascal Raybaud

S1. X-ray transmission as a function of energy with fused silica window thickness

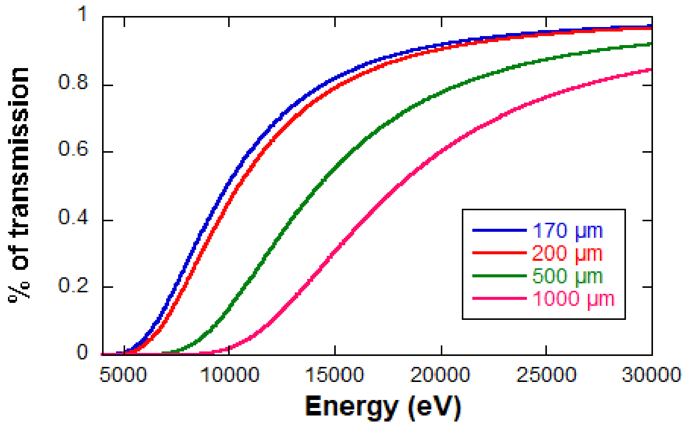

Figure S1 X-ray transmission as a function of energy depending on the thickness (170, 200, 500 and 1000 µm) of the fused silica window.

S2. Additional schemes of the *operando* XAS photocatalytic cell

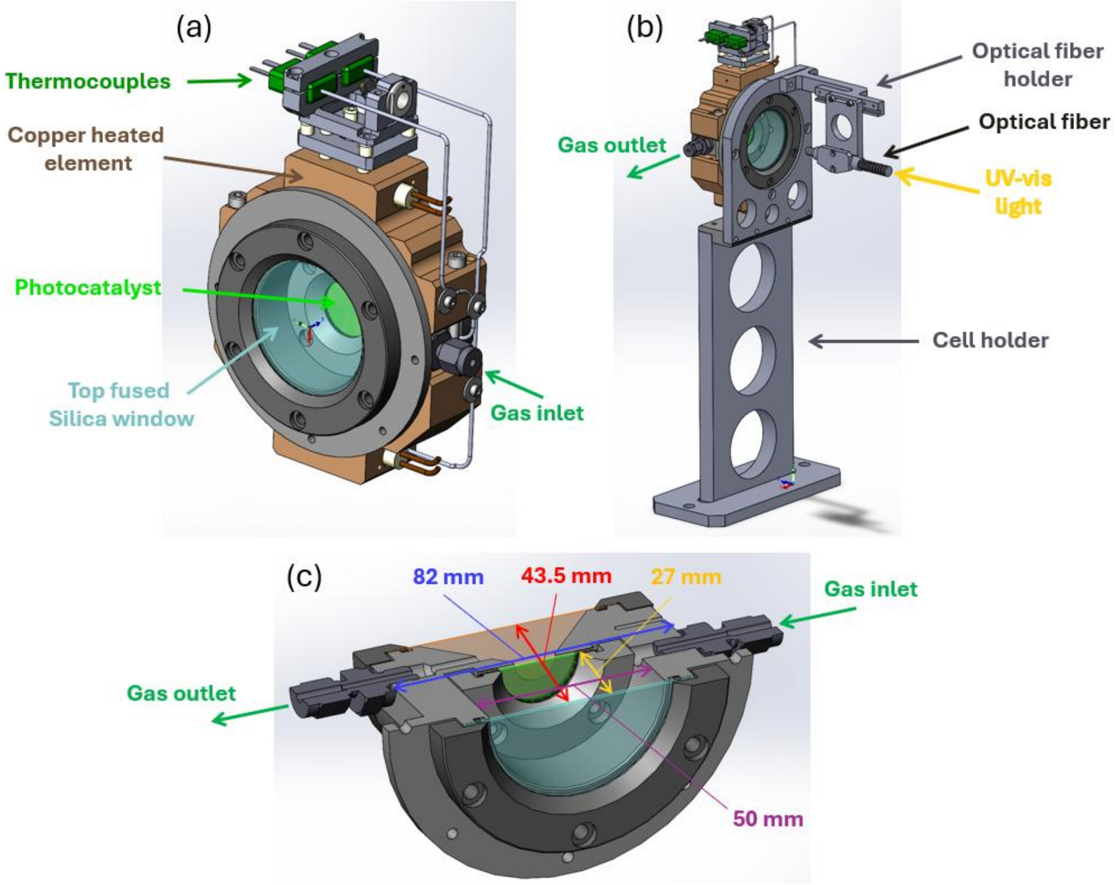

Figure S2 Schematics of (a) the operando XAS photocatalytic cell, (b) the vertically mounted cell with the optical fiber positioned perpendicular to the photocatalyst surface and (c) section of the cell with its main measures.

S3. Precautions for XAS data acquisition

Precautions to avoid any evolution of the Mo species induced by X-ray radiations are taken: the X-ray beam is attenuated by a 2 mm thick glass at the entrance of the first ionization chamber measuring I_0, and the analysed area of the sample is changed every 30 min (with an automated motor moving the cell) throughout the test. When such precautions are not taken, XANES spectra exhibit very strong evolution throughout the test, due to beam damage (**Error! Reference source not found.**). When such precautions are taken, no beam damage is observed, otherwise a shift toward lower energies at 0.8 normalized absorption should be observed when changing to a new position that has never received X-rays before, which is not the case (**Error! Reference source not found.**).

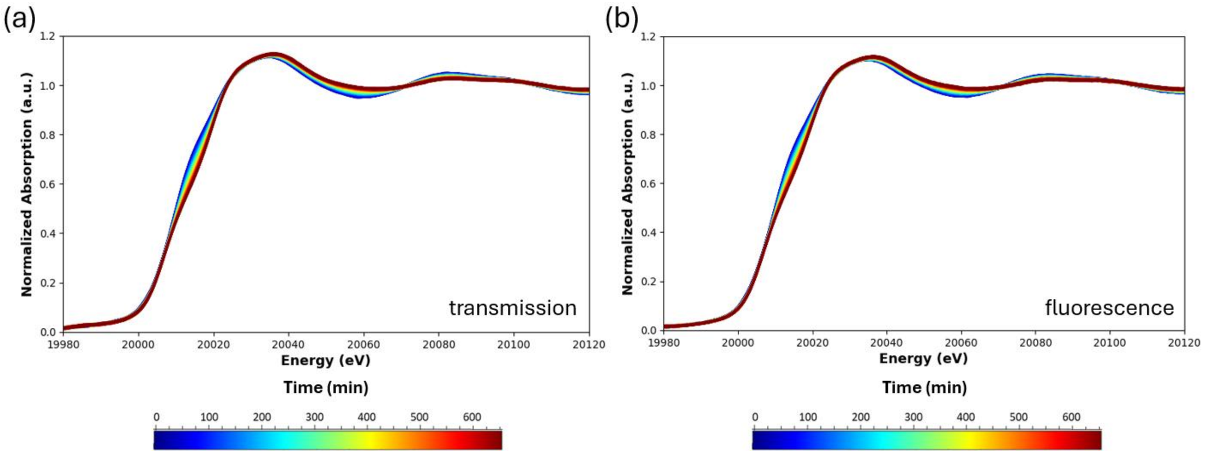

Figure S3 3%MoO_xS_y(20°C)/TiO_2 normalised Mo K-edge XANES accumulated throughout the whole operando photocatalytic test in (a) transmission and (b) fluorescence without any precaution regarding the intensity of the X-ray beam and the area analysed.

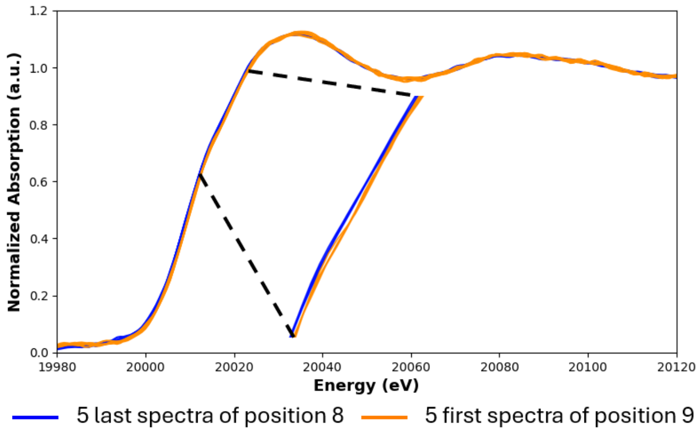

Figure S4 3%MoO_xS_y(20°C)/TiO_2 normalised Mo K-edge XANES spectra of the 5 last transmission spectra registered at position 8 (blue spectra) and of the 5 first transmission spectra registered at position 9 (orange spectra). 5 spectra are acquired in 75 s. XAS measurements are switched to position 9 at ~270 min of test.

S4. Photocatalytic activity comparison

The photocatalytic activity of 3%Mo(20°C)/TiO_2 and 3%Mo(350°C)/TiO_2 is compared in Table S1 over 4.2h of irradiation on the ROCK set-up and 5.6h or irradiation on the IFPEN set-up. The time of irradiation to report the data was chosen as the shortest time of test available on the ROCK set-up (4.2h). Due to the different dead-volumes on the two set-ups (~20 mL for ROCK vs ~30 mL for IFPEN), the time to reach the maximum of CO_2 conversion (CO + CH_4 rate of production) is of ~270 min on the ROCK set-up and ~360 min on the IFPEN set-up (**Error! Reference source not found.**), which is why the time of irradiation to report data for the IFPEN set-up was chosen at 5.6h (= 4.2 x (360/270)).

The differences observed in CO and CH_4 production between the two set-ups could arise from the differences that are specific to the two set-ups: irradiation source and condition, working pressure, etc…

Table S1 Photocatalytic tests results: electrons consumption and electronic selectivity for CH_4, CO, and H_2 production over 4.2h of continuous or alternation illumination (ROCK set-up) or 5.6h of continuous illumination (IFPEN set-up), electronic selectivity toward carbonaceous products for 3%Mo(20°C)/TiO_2 and 3%Mo(350°C)/TiO_2.

		e- for CH_4 production (electronic selectivity)	e- for CO production (electronic selectivity)	e- for H_2 production (electronic selectivity)	Electronic selectivity toward carbonaceous products
ROCK set-up	3%Mo(20°C)/TiO_2 4.2h continuous irradiation	1692 µmol.m^{-2} 10.72 µmol.g^{-1} (81.6%)	339 µmol.m^{-2} 2.15 µmol.g^{-1} (16.3%)	43 µmol.m^{-2} 0.27 µmol.g^{-1} (2.1%)	97.9%
	3%Mo(350°C)/TiO_2 4.2h *alternating* irradiation	262 µmol.m^{-2} 1.66 µmol.g^{-1} (54.8%)	127 µmol.m^{-2} 0.80 µmol.g^{-1} (26.6%)	89 µmol.m^{-2} 0.56 µmol.g^{-1} (18.6%)	81.4%
IFPEN set-up	3%Mo(20°C)/TiO_2 5.6h continuous irradiation	2392 µmol.m^{-2} 16.98 µmol.g^{-1} (96.4%)	32 µmol.m^{-2} 0.23 µmol.g^{-1} (1.3%)	58 µmol.m^{-2} 0.41 µmol.g^{-1} (2.3%)	97.7%
	3%Mo(350°C)/TiO_2 5.6h continuous irradiation	520 µmol.m^{-2} 3.69 µmol.g^{-1} (91.6%)	20 µmol.m^{-2} 0.14 µmol.g^{-1} (3.5%)	28 µmol.m^{-2} 0.20 µmol.g^{-1} (4.9%)	95.1%

S5. Experiments artefact linked to the dead-volume

The increase of converted CO_2 rate (**Error! Reference source not found.**) is an artefact of the process due to the dead-volume (volume between the catalytic bed and the µGC) and slow flow rate (0.3 mL.min^{-1}). Indeed, the dead-volume differences in the two set-ups (~20 mL for ROCK vs ~30 mL for IFPEN) explain the shift in time to reach the maximum of converted CO_2 (~270 min for ROCK vs ~360 min for IFPEN). To further emphasize the artefact effect, the same phenomenon happens when testing a photocatalyst of different nature (1%$_{wt}$Pt/PC500 TiO_2) on the IFPEN set-up: the maximum of production is still reached at ~360 min (Figure S5a) (Dankar *et al.* 2023b). Yet, on a set-up with much smaller dead-volume (0.42 mL (Dankar *et al.* 2023 a)) and much higher flow rate (5 mL.min^{-1}), such increase of CH_4 production is not observed at all (mass spectrometer detection), the maximum of production is reached instantaneously (Figure S5b) (Dankar *et al.* 2023b).

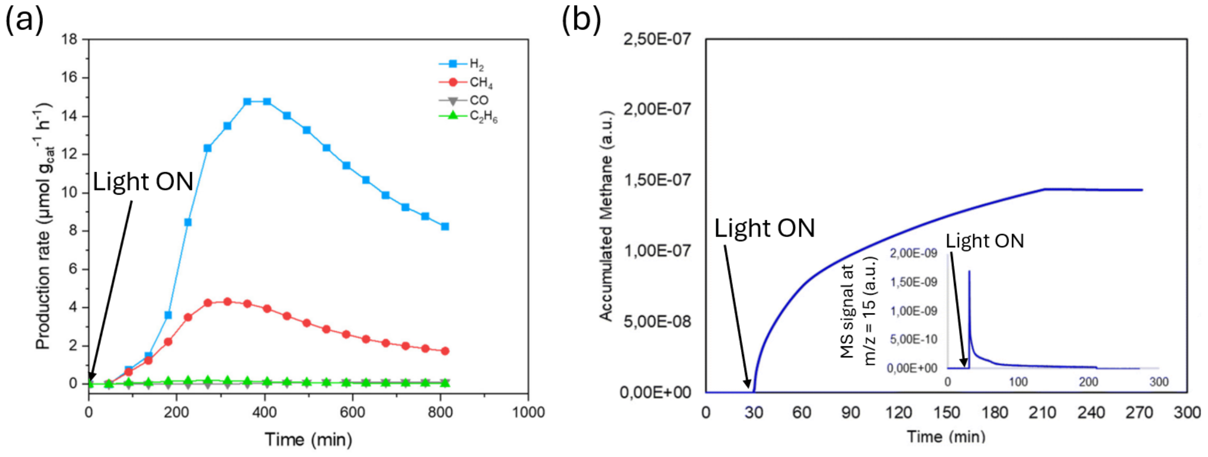

Figure S5 (a) Products evolution during the photocatalytic reduction of CO_2 with water under UV-Vis irradiation on the set-up used at IFPEN for 1%$_{wt}$Pt/PC500 TiO_2 and (b) gas phase acquisition of methane recorded using a mass spectrometer at m/z = 15 in a different set-up (in *operando* IR spectrometer in LCS Caen with 0.42 mL dead volume) during CO_2 photoreduction with the same 1%$_{wt}$Pt/PC500 TiO_2 material. (Dankar *et al.* 2023b)

Additionnally, any change in the production in the reactor is seen by the µGC with a delay of ~10 min (time before observing any production after switching on the irradiation (**Error! Reference source not found.**e), therefore after the maximum of production is reached, any change in the production should be observed quasi-simultaneously on the µGC.

S6. Performances under continuous irradiation vs alternating irradiation

The evaluation of the photocatalytic performances is not drastically impacted by the use of alternating UV-visible irradiation (180 s ON/ 180 s OFF) compared to continuous UV-visible irradiation, a maximum factor of 2 on the rate of formation can be expected, as it is demonstrated for the 3%MoO_xS_y(20°C)/TiO_2 photocatalyst (Figure S6).

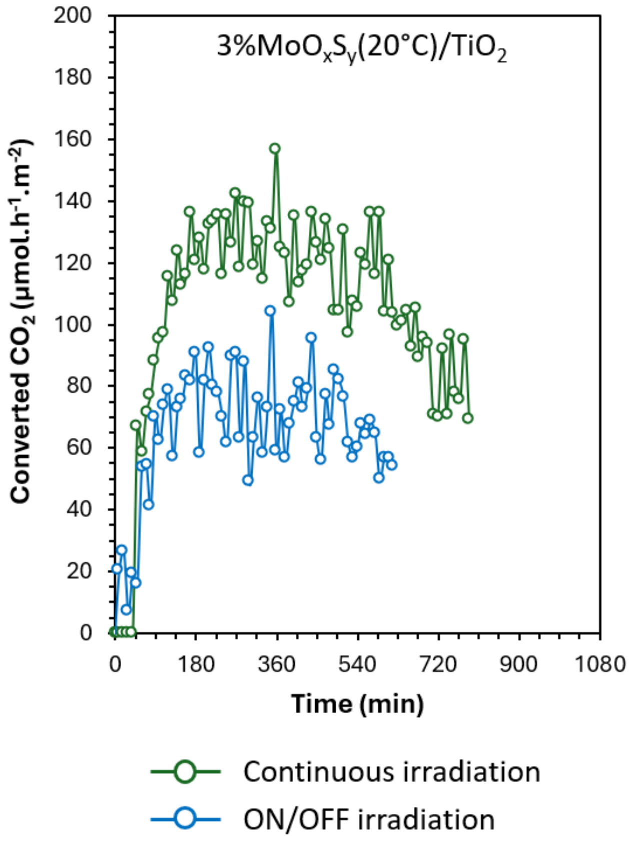

Figure S6 Converted CO_2 (in µmol.h^{-1}.m^{-2}) evolution in the ROCK set-up for 3%MoO_xS_y(20°C)/TiO_2 under continuous UV-visible light irradiation (green circles) and alternating UV-visible irradiation: 180 s ON / 180 s OFF (blue circles).

S7. CO rate of formation under alternating UV-visible irradiation

CO rate of formation increases up to ~200 min and then reaches a plateau of ~35 µmol.h^{-1}.m^{-2} (Figure S7a). This evolution does not match with the observed evolution of Mo species throughout the photocatalytic test which evolve up to ~385 min (Figure S7b).

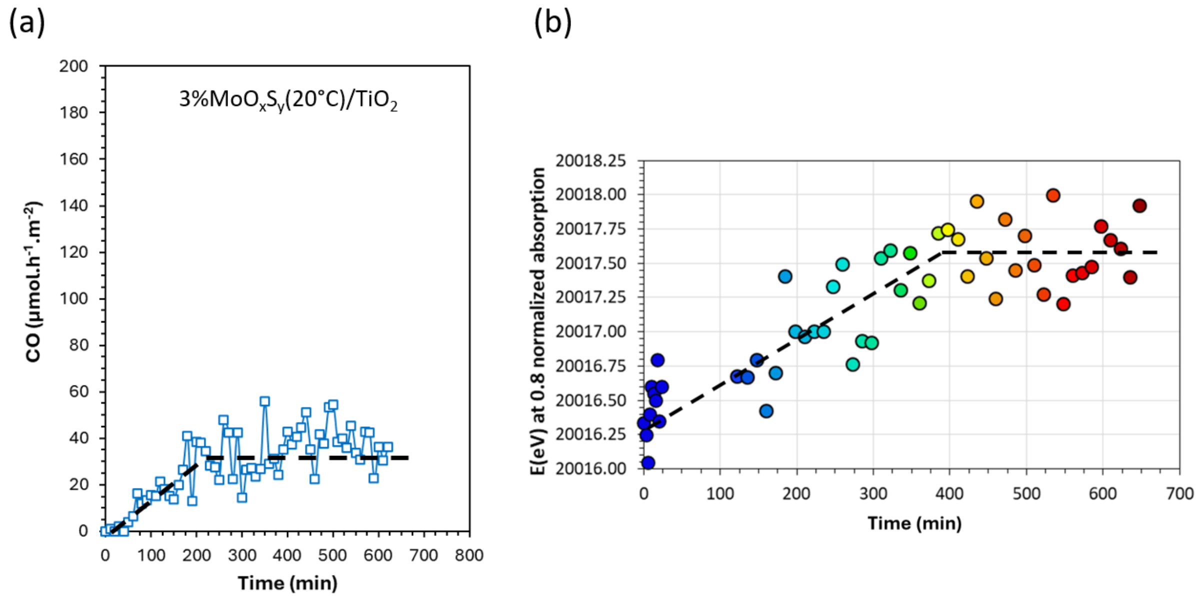

Figure S7 (a) CO formation rate (in µmol.h^{-1}.m^{-2}) evolution in the ROCK set-up for 3%MoO_xS_y(20°C)/TiO_2 under alternating UV-visible irradiation: 180 s ON / 180 s OFF. (b) Mo K-edge energy (in eV) at 0.8 of normalized absorption (XANES) accumulated in transmission evolution in time for 3%MoO_xS_y(20°C)/TiO_2. Dashed lines represent a guide to the eyes to observe trends (not based on mathematical fitting).

S8. *Operando* XAS additional data

Merging the first and last 10 XANES spectra of 3%MoO_xS_y(20°C)/TiO_2 emphasizes the shift in energy that occurred during the *operando* XAS photocatalytic experiment under alternating UV-visible irradiation (Figure S8).

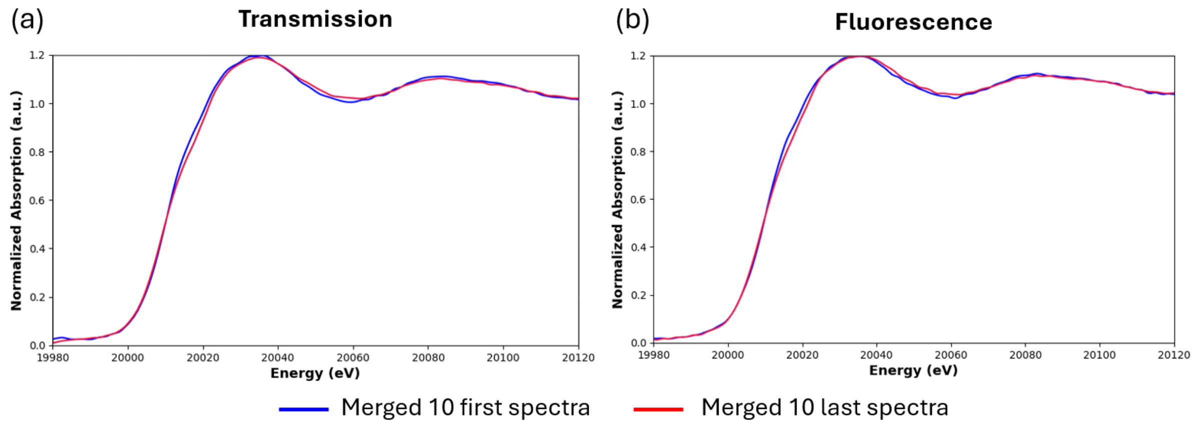

Figure S8 3%MoO_xS_y(20°C)/TiO_2 normalised and merged 10 first and last Mo K-edge XANES spectra accumulated in (a) transmission and (b) fluorescence during the *operando* photocatalytic test. 10 spectra are acquired in 150 s.

The changes observed on the XANES spectra are also slightly reflected on the EXAFS spectra for both transmission and fluorescence (Figure S9), which may indicate that the local geometry around the Mo atoms is affected during the photocatalytic test as well.

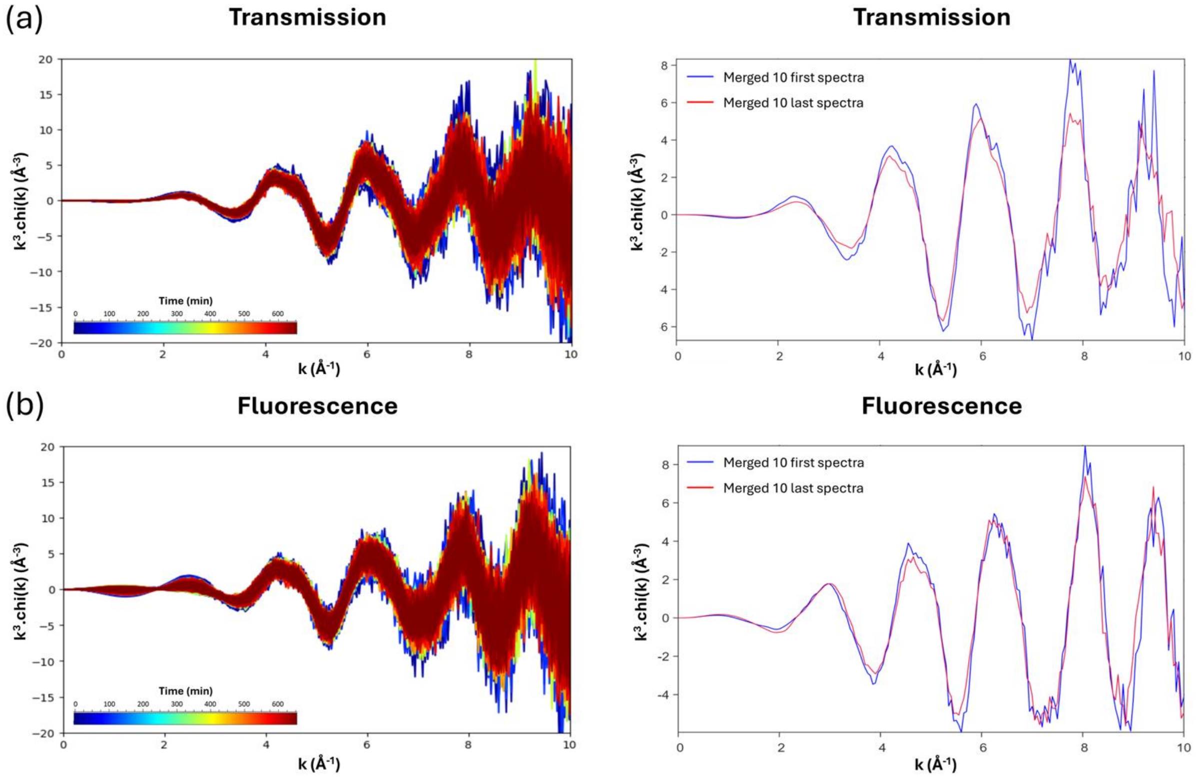

Figure S9 3%MoO_xS_y(20°C)/TiO_2 Mo K-edge EXAFS (k^3-weighted $\chi(k)$) accumulated throughout the whole *operando* photocatalytic test and merged 10 first and 10 last spectra in (a) transmission and (b) fluorescence. 10 spectra are acquired in 150 s.
